# Supplementary material for: High Rates of COVID-19 Vaccine Hesitancy and Its Association with Conspiracy Beliefs: A Study in Jordan and Kuwait among Other Arab Countries
Source: Vaccines (Basel). 2021 Jan 12;9(1):42. doi: 10.3390/vaccines9010042 (PMC7826844; doi:10.3390/vaccines9010042)
Supplement: Supplementary file 1 [file vaccines-09-00042-s001.pdf]

Supplementary S1

Consent form and questionnaire translated to English (the original form in Arabic is provided below).

SARS-CoV-2 and COVID-19 vaccine: Level of knowledge in Jordan and other Arab countries

This questionnaire has been prepared to measure the extent of knowledge in Jordan and other Arab countries about the prospective coronavirus disease 2019 (COVID-19) vaccine. The information provided to you through this questionnaire will be used for research purposes only, and the data will be treated with complete confidentiality and privacy.

Participation in this survey is completely voluntary.

The average time to complete the questionnaire is only 3 minutes.

Thank you very much for agreeing to take part in this survey.

Section #1

1. Age

………………….

2. Sex

Male

Female

3. The country in which you reside:

United Arab Emirates

Jordan

Bahrain

Algeria

Saudi Arabia

Sudan

Somalia

Iraq

Kuwait

Morocco

Yemen

Tunisia

Comoros

Djibouti

Syria

Oman

Palestine

Qatar

Lebanon

Libya

Egypt

Mauritania

4. Monthly income of the household in case you are resident in Jordan

Less than 500 Jordanian dinars.

500 – 1000 Jordanian dinars.

More than 1000 Jordanian dinars.

5. Educational level

High school or less

Undergraduate degree (diploma, BSc degree)

Postgraduate degree (MSc, PhD degrees)

6. Do you suffer from any chronic diseases (such as diabetes, hypertension or heart disease)?

Yes

No

7. Have you or any of your family members had COVID-19?

Yes

No

Section #2

8. What is your belief about the origin of the current coronavirus in humans?

Natural source from animals.

Man-made virus and part of a conspiracy plot.

9. Do you think the current coronavirus was man-made to force everyone to get vaccinated?

Yes

No

10. Will you get the coronavirus vaccine when available?

Yes

No

11. Have you had or are you going to have the influenza vaccine?

Yes

No

12. Do you oppose vaccination altogether?

Yes

No

13. Do you think that coronavirus vaccine will be a way of implanting people with microchips in order to control humans?

Yes

No

14. COVID-19 vaccines will lead to infertility

Yes

No

15. Do you think it is acceptable for the government to force everyone to get coronavirus vaccine?

Yes

No

16. What is your main source of information about coronavirus vaccine?

TV programs and news releases

Social media platforms (Facebook, Twitter, Instagram, WhatsApp)

Medical doctors, scientists and scientific journals

YouTube

For each statement, please indicate how much you disagree or agree by selecting the appropriate number:

**Item**	*Strongly Disagree*	*Disagree*	*Somewhat Disagree*	*Neutral*	*Somewhat Agree*	*Agree*	*Strongly Agree*
17. COVID-19 vaccine safety data is often fabricated.							
18. Immunizing children is harmful, and this fact is covered up.							
19. Pharmaceutical companies cover up the dangers of COVID-19 vaccines.							
20. People are deceived about COVID-19 vaccine efficacy.							
21. Vaccine efficacy data is often fabricated.							
22. People are deceived about vaccine safety.							
23. The government is trying to cover up the link between vaccines and autism.							

Thank you very much for participating in the survey

لقاح فيروس كورونا ومرض كوفيد ٢٠١٩: مستوى المعرفة في الأردن والدول العربية

تم إعداد هذا الاستبيان لقياس مدى المعرفة في الأردن والدول العربية حول لقاح فيروس كورونا المستجد كوفيد ١٩ .سيتم استخدام المعلومات المقدمة لك من خلال هذا الاستبيان لأغراض بحثية فقط وسيتم التعامل مع البيانات بسرية وخصوصية تامة

المشاركة في هذا الاستبيان طوعية تمامًا

معدل وقت استكمال الاستبيان هو ٣ دقائق فقط

شكراُ جزيلاُ للموافقة على المشاركة في هذا الاستبيان

العمر: ………

الجنس:

ذكر

أنثى

الدولة التي تسكن / تسكنين فيها:

الإمارات العربية المتحدة

الأردن

البحرين

الجزائر

السعودية

السودان

الصومال

العراق

الكويت

المغرب

اليمن

تونس

جزر القمر

جيبوتي

سوريا

عمان

فلسطين

قطر

لبنان

ليبيا

مصر

موريتانيا

الدخل الشهري للأسرة في حال كنت تعيش في الأردن

أقل من 500 دينار

بين 500 و 1000 دينار

أكثر من 1000 دينار

المستوى التعليمي:

ثانوية عامة أو أقل

دبلوم أو بكالوريوس

ماجستير أو دكتوراه

هل تعاني من أي أمراض مزمنة (مثل السكري، أو الضغط، أو أمراض في القلب)؟

نعم

لا

هل أُصبت أنت أو أحد أفراد أسرتك بكوفيد ١٩؟

نعم

لا

ما هو اعتقادك بشأن مصدر فيروس كورونا في البشر؟

مصدر طبيعي من الحيوانات

فيروس مُصنع وجزء من مؤامرة

هل تعتقد أن فيروس كورونا تم تصنيعه لإجبار الجميع على أخذ اللقاح؟

نعم

لا

هل ستأخذ لقاح الكورونا عند توفره؟

نعم

لا

هل أخذت أو ستأخذ لقاح الانفلونزا؟

نعم

لا

هل أنت من معارضين اللقاحات إجمالاً؟

نعم

لا

هل تعتقد أن لقاح كوفيد ١٩ سيكون وسيلة لحقن الناس بشرائح مجهرية للسيطرة على البشر؟

نعم

لا

سيؤدي لقاح كوفيد ١٩ إلى العُقم

نعم

لا

هل تعتقد أنه من المقبول أن تُجبر الحكومة الجميع على أخذ لقاح كورونا؟

نعم

لا

ما هو المصدر الرئيسي لمعلوماتك حول اللقاح؟

برامج التلفاز و النشرات الإخبارية

وسائل التواصل الاجتماعي (Facebook, Twitter, Instagram, ،WhatsApp)

الأطباء والعلماء والمجلات العلمية المتخصصة

يوتيوب (YouTube)

لكل عبارة ، يُرجى توضيح مدى عدم موافقتك أو موافقتك عن طريق تحديد الرقم المناسب:

	أرفض بشدة	أعارض	أعارض إلى حدٍ ما	مُحايد	أوافق إلى حدٍ ما	أوافق	أوافق بشدة
غالبًا ما تكون بيانات سلامة لقاح كوفيد مُزيفة	☐	☐	☐	☐	☐	☐	☐
تطعيم الأطفال ضار وهذه الحقيقة محجوبة عن الناس	☐	☐	☐	☐	☐	☐	☐
تُخفي شركات الأدوية عن الناس مخاطر لقاح كوفيد ١٩	☐	☐	☐	☐	☐	☐	☐
يتم خداع الناس بشأن فعالية لقاح كوفيد ١٩	☐	☐	☐	☐	☐	☐	☐
غالبًا ما تكون بيانات فعالية اللقاح مُزيفة	☐	☐	☐	☐	☐	☐	☐
يتم خداع الناس بشأن سلامة اللقاحات	☐	☐	☐	☐	☐	☐	☐
تحاول الحكومة التستر على الصلة بين اللقاحات وأمراض أُخرى مثل التوحد	☐	☐	☐	☐	☐	☐	☐

شكراً جزيلاً على مُشاركتكم في الاستبيان
